# Isolated Neisseria meningitidis-associated endophthalmitis in an immunocompetent host: case report and literature review

**DOI:** 10.1099/acmi.0.000901.v3

**Published:** 2025-03-07

**Authors:** Calvin Ka-Fung Lo, Lauren Hughes, Cole Schonhofer, William R. Bowie, Iain McCormick, Andrew Kirker

**Affiliations:** 1 Department of Pathology and Laboratory Medicine, University of British Columbia, Vancouver, British Columbia, Canada; 2 Department of Ophthalmology and Visual Sciences, University of British Columbia, Vancouver, British Columbia, Canada; 3 Division of Infectious Diseases, University of British Columbia, Vancouver, British Columbia, Canada; 4 Division of General Internal Medicine, University of British Columbia, Vancouver, British Columbia, Canada

**Keywords:** complement deficiency, endophthalmitis, meningococcus, *Neisseria meningitidis*, serogroup

## Abstract

**Introduction.***Neisseria meningitidis* is a Gram-negative diplococcus with significant infectious sequelae, including meningitis and disseminated meningococcal bloodstream infection. Rarely has it been reported in the context of endophthalmitis without central nervous system involvement. We report a clinical case of isolated meningococcal endophthalmitis in an immunocompetent patient and present a literature review on published cases, including treatment regimens and clinical outcomes.

**Case Presentation.** A 51-year-old male with no significant medical history presented to the emergency department with acute vision loss in the right eye after returning from Mexico. Ophthalmic examination was consistent with endophthalmitis, presumed to be endogenous in the absence of recent ocular trauma or surgery. Vitreous culture was positive for growth of Gram-negative diplococci, subsequently identified as *N. meningitidis*. Blood and cerebrospinal fluid cultures were negative for growth of similar or implicative pathogens. There was no evidence of disseminated meningococcal infection; imaging did not demonstrate any drainable collections or sequelae of extension into the central nervous system. The patient was treated with intravitreal antibiotics as well as topical steroids and antibiotics. In addition, he completed a 2-week course of systemic antibiotics. Visual outcome was unfortunately poor.

**Conclusion.** This case illustrates a rare case of isolated endophthalmitis secondary to meningococcus, presumably from a nasopharyngeal source. In these clinical scenarios, clinicians should perform a thorough evaluation for predisposing immunodeficiencies.

## Data Summary

All data generated or analysed during this study are included in this published article (and its supplementary information files). See Tables1  2, as well as File S1 for data extracted from eligible articles within our literature review and File S2 for the extracted list of all English-language PubMed articles identified with our search phrase.

**Table 1.** Summary of patients with documented visual outcomes (*n*=25 cases)

Patient characteristic	Value
**Median age (range, IQR)**	17 years (0–82, 7–27.5)
**Sex**	
Male	15 (60 %)
Female	10 (40 %)
**Comorbidities**	
None	19 (76 %)
Diabetes mellitus	1 (4 %)
Hypertension	2 (8 %)
Previous ocular surgeries and presence of a filtering bleb	4 (16 %)
**Prodrome**	
Present	21 (84 %)
Average duration	6.4 days
**Prodromesymptoms**	
Fever	11 (44 %)
Rash	12 (48 %)
Myalgia/Arthralgia	12 (48 %)
Malaise	6 (24 %)
Vomiting/diarrhoea	5 (20 %)
URI symptoms	5 (20 %)
**Other systemic manifestations**	
Bacteraemia	5 (20 %)
Pericarditis	1 (4 %)
DIC	1 (4 %)
Septic joint	2 (8 %)
**Eyes affected**	
One eye	20 (80 %)
Both eyes	5 (20 %)
**Presenting visual acuity**	
Light perception or worse	11 (44 %)
Hand movements or counting fingers	8 (32 %)
Better than counting fingers	2 (8 %)
Not stated	4 (16 %)
**Initial diagnosis**	
Endophthalmitis	13 (52 %)
Conjunctivitis	3 (12 %)
Uveitis	8 (32 %)
Retinoblastoma	1 (4 %)
***N.meningitidis*** **isolated from:**	
Vitreous aspiration/biopsy	16 (64 %)
Anterior chamber sample	3 (12 %)
Blood	5 (20 %)
Skin scrapings	1 (4 %)
**Diagnostic method**	
Culture or not otherwise stated	18 (72 %)
PCR	4 (16 %)
Culture+PCR	2 (8 %)
16 s rRNA	1 (4 %)
**Serogroup**	
Not reported	13 (52 %)
C	10 (40 %)
W135	1 (4 %)
Y	1 (4 %)
**Antibiotic route**	
Intravenous	25 (100 %)
Intravitreal	19 (76 %)
Subconjunctival	5 (20 %)
Topical	18 (72 %)
**Intravitreal antibiotics used (** ***n*** **=19)**	
Ceftazidime and vancomycin	9 (36 %)
Amikacin and vancomycin	5 (20 %)
Gentamicin and cefazolin	1 (4 %)
Penicillin	1 (4 %)
Vancomycin alone	1 (4 %)
**Duration of systemic antibiotic treatment (** ***n*** **=25)**	
Not stated	12 (48 %)
≤2 weeks	9 (36 %)
>2 weeks	4 (16 %)
**Steroids**	
Topical	16 (64 %)
Systemic	8 (32 %)
Intravitreal	4 (16 %)
Subconjunctival	3 (12 %)
None	8 (32 %)
**Mydriatics**	16 (64 %)
**Surgical procedures**	
Vitrectomy	16 (64 %)
Enucleation	2 (8 %)
None	6 (24 %)
**Outcome**	
‘Non-functional’ outcome	14 (56 %)
‘Functional’ outcome	10 (40 %)
Vision<20/200 before infection	1 (4.%)

“‘Functional’ outcome is defined as visual acuity> greater than 20/200 in at least one affected eye at last recorded clinical follow-up. ‘Non-functional’ outcome is defined as visual acuity less than <20/200 in affected eye or both affected eyes.

IQRinterquartile ratioURIupper respiratory infection

**Table 2.** Comparison between patients with functional versus non-functional recovery post-endophthalmitis

Patient variable	Functional outcome (*n*=10)	Non-functional outcome (*n*=14)
**Median age**	13	19.5
**Sex**		
Male	6 (60%)	8 (57.1%)
Female	4 (40%)	6 (42.9%)
**Comorbidities**		
None	8 (80%)	11 (78.6%)
Filtering bleb	2 (20%)	2 (14.3%)
Diabetes		1 (7.1%)
**Time fromocular symptom onset to systemic or Intravitreal antibiotics (average±** **sd** **)**	3.9±2.6 Days(*n*=8)Not stated(*n*=2)	4.9±5.8 Days(*n*=13)Not stated(*n*=2)
**Visualacuity atpresentation**		
Better than LP	4 (40%)	6 (42.9 %)
LP or worse	4 (40%)	6 (42.9 %)
Not stated	2 (20%)	2 (14.3 %)
**Initial misdiagnosis**	4 (40%)	8 (57.1 %)
**Time frompresentation to systemic orintravitreal antibiotics (average±** **sd** **)**	1.1±1.6 days(*n*=8)Not stated(*n*=2)	2.5±4.9 days(*n*=13)Not stated(*n*=1)
**Antibioticroute**		
IV only	1 (10 %)	2 (14.3 %)
IV+T	2 (20 %)	
IV+IVT		3 (21.4 %)
IV+IVT+T	3 (30 %)	9 (64.3 %)
IV+IVT+ SCJ	1(10 %)	
IV+SCJ+T	1 (10 %)	1 (7.1 %)
IV+SCJ+T+IVT	2 (20 %)	
**Systemic antibiotic duration**		
Not stated	5 (50 %)	6 (42.9 %)
≤2 weeks	5 (50 %)	4 (28.6 %)
>2 weeks		4 (28.6 %)
**Steroidsused**	7 (70 %)	9 (64.3 %)
**Steroidroute (** ***n*** **=16)**		
T	3 (30 %)	3 (21.4 %)
IVT		1 (7.1 %)
T+Systemic	2 (20 %)	1 (7.1 %)
T + IVT		1 (7.1 %)
T+SCJ		1 (7.1 %)
T+Systemic+ IVT		2 (14.2 %)
T+Systemic+ SCJ	2 (20 %)	
**Mydriaticsused**	7 (70 %)	9 (64.3 %)
**Vitrectomyperformed**	5 (50 %)	10 (71.4 %)

“‘Functional’ outcome: visual acuity>20/200 in at least one affected eye at last recorded follow-up. ‘Non-functional’ outcome: visual acuity<20/200 in affected eye or both affected eyes. LP Light perception, IV intravenous, T topical, IVT intravitreal, SCJ subconjunctival, STD Standard Deviation.

IVintravenousIVTintravitrealLPlight perceptionSCJsubconjunctivalTtopical

## Introduction

*Neisseria meningitidis* is a Gram-negative diplococcus under the *Neisseriaceae* family, often referred to as meningococcus [1]. Amidst its commensal relatives (e.g. *Neisseria subflava* and *Neisseria polysaccharea*), *N. meningitidis* is a pathogenic species with implications for significant invasive disease. It is an obligate human pathogen with a reservoir generally residing in the nasopharynx. Up to 10% of individuals are colonized, with a higher predilection for adolescents and young adults. It appears as round, colourless-to-grey, non-pigmented, non-haemolytic colonies upon growth on solid agar media [2]. Depending on the extent of produced polysaccharide, colonies may appear mucoid. It grows generally well on blood, chocolate and Mueller–Hinton agars. Predating molecular diagnostic methods, confirmation used to involve a positive oxidase test and the presence of specific carbohydrate fermentation. *N. meningitidis* metabolizes glucose and maltose but fails to metabolize sucrose or lactose [2].

Although most carriers are asymptomatic, *N. meningitidis* can cause a wide spectrum of infectious disease, from mucosal infections such as sinusitis and otitis to invasive disease such as meningococcaemia, septic arthritis and central nervous infections (e.g. meningitis) [1]. It transmits via direct contact with respiratory secretions or inhalation of large respiratory droplets. Here we present a case of *N. meningitidis* endophthalmitis in an immunocompetent patient. We performed a literature review of endophthalmitis cases associated with *N. meningitidis* without clinical evidence of meningitis as well as a review of risk factors, microbiologic workup and clinical sequelae.

## Case presentation

A 51-year-old male presented to the emergency department on 23 January 2023 with a 3 day history of progressive vision loss, pain and redness involving the right eye. He had no past ocular history, including no preceding ophthalmic procedures. The patient was otherwise healthy with a past medical history significant only for remote appendectomy. His immunizations were up to date; he was also vaccinated against Hepatitis A and Diphtheria–Pertussis–Tetanus in 1997.

The patient had recently travelled to a resort in Mexico between 11 and 18 January and had acquired a minor laceration on his forehead after falling in the bathroom on 13 January (i.e. 7 days prior to symptom onset). There was no additional history of ocular or head trauma. On detailed review of systems, the patient did not report any sick contact exposures nor obvious animal or insect bites. He denied significant headaches, bleeding gums, fevers, chills, vomiting, cough, rhinitis, sore throat and urinary symptoms. Prior to returning from Mexico, he did report episodes of non-bloody diarrhoea that started on 17 January before ocular symptom onset; his diarrhoea resolved spontaneously by 20 January. On 21 January, following the resolution of his diarrhoea, the patient developed right eye pain, accompanied by ‘reddish-brown floaters’ and progressive vision loss.

In terms of potential risk factors, there was no history of intravenous drug use. He had been sexually abstinent since summer 2020. Previously, he engaged in both oral and penile–vaginal sex with female partners only, with inconsistent use of barrier contraceptives. He had never been tested for sexually transmitted infections, other than a documented skin swab positive for varicella zoster virus (no lesion site listed) in November 2015.

Given the patient’s concerning presentation with profound vision loss, he was urgently evaluated by the ophthalmology service. Best corrected visual acuity was light perception in the right eye (OD) and 20/20 in the left eye (OS). Intraocular pressures were 15 mmHg OD and 16 mmHg OS. Anterior segment examination OD revealed eyelid oedema and erythema, 360° of subconjunctival haemorrhage and significant corneal oedema. There was also a layered hypopyon and clotted blood in the anterior chamber, with no view of the fundus. B-scan ultrasound showed an attached retina with highly echogenic vitreous material, suggestive of dense vitritis. Examination OS was within normal limits.

A vitreous tap was performed OD for culture, followed by administration of intravitreal antibiotics (ceftazidime 2 mg and vancomycin 1 mg) and steroids (dexamethasone 0.4 mg). In addition, the patient was treated with topical steroids (prednisolone acetate 1% eye drops every 2 h) and mydriatics (atropine twice daily). Topical antibiotics (i.e. moxifloxacin four times per day) were added 4 days later (27 January) and discontinued on 6 February.

On physician examination, the patient was clinically alert and oriented with an appropriate response to commands. Although mildly icteric, he was haemodynamically stable with no signs of distress. Vital signs showed a temperature of 37.4 °C, blood pressure of 104/69, heart rate of 100 and oxygen saturation of 98% on room air. His physical examination was unremarkable for any stigmata of infective endocarditis, nor any concerning signs of meningismus. There was an absence of genital ulcerations, discharge and inguinal lymphadenopathy. There was no presence of petechiae or purpura across the body or distal extremities, nor evidence of arthritis or joint effusions.

Initial blood work showed leukocytosis of 15.2×10^9^ l^−1^ (reference range 4.0–11.0×10^9^ l^−1^) with neutrophils of 11.4×10^9^ l^−1^ (reference range 4.0–11.0×10^9^ l^−1^), mild normocytic anaemia with haemoglobin of 123 gl^−1^ [mean corpuscular volume (MCV) volume of 100] (reference range 135–170 gl^−1^ with MCV 80–100) and low platelets of 143×10^9^ l^−1^ (reference range 150–400×10^9^ l^−1^). He had a C-reactive protein of 50.3 mg l^−1^ (reference range<3.1 mg l^−1^). His liver enzymes were reflective of a cholestatic pattern: alkaline phosphatase (ALP) 144 µl^−1^, gamma-glutamyl transferase (GGT) 161 µl^−1^ with total bilirubin of 46 µmol l^−1^ and alanine transferase (ALT) of 56 µl^−1^ (reference ranges: ALP 30–135 µl^−1^; GGT 15–80 µl^−1^; total bilirubin<20 µmol l^−1^; ALT 10–55 µl^−1^). This was thought to be secondary to his underlying alcohol use (reportedly drinking up to 20 units of alcohol per week); on repeat measure 2 days into admission, his enzymes largely normalized: ALP 123, GGT 131 with normal total bilirubin of 13 µmol l^−1^ and ALT of 59 µl^−1^ (his abdominal ultrasound revealed moderate hepatic steatosis with evidence of small gallstones up to 4 mm, without evidence of biliary duct dilatation). Furthermore, he had laboratory evidence suggestive of acute kidney injury thought to be secondary to hypovolaemia [serum creatinine (Cr) of 234 µmol l^−1^, estimated glomerular filtration rate (eGFR) 27 ml min^−1^, which improved to serum Cr 120, eGFR 60 ml min^−1^ after intravenous fluids and improved oral intake; reference range: Cr 60–115 µmol l^−1^; GFR>59 ml min^−1^]. Urinalysis showed moderate leukocytes, negative nitrites and moderate haemoglobin and presence of protein (0.3 gl^−1^). There were no ketones, bilirubin or glucose noted, and urine microscopy did not detect any casts or crystals.

## Diagnosis, treatment and follow-up

Vitreous culture smear showed 4+ polymorphs with 2+ Gram-negative diplococci and 1+(rare) Gram-positive cocci. Culture media had light growth of Gram-negative diplococci (the Gram-positive cocci failed to grow, which was thought either to be due to skin contaminant at low inoculum or that the Gram-positive cocci were actually Gram-negative cocci that did not properly decolourize during Gram stain processing). MALDI-ToF MS subsequently identified *N. meningitidis*. Given the presence of *N. meningitidis* in a sterile site and concerns for irreversible vision loss, the patient had peripheral blood cultures collected and subsequently was started on intravenous ceftriaxone 2000 mg q12h and a loading dose of intravenous vancomycin 1500 mg. Close contacts (including his household members) and ophthalmology colleagues who obtained the vitreous sample prior to antimicrobial administration were offered ciprofloxacin 500 mg PO (single dose) for chemoprophylaxis; subsequently, no other chemoprophylaxis cases were identified during hospitalization, as the patient was already started on at least 24 h of effective antimicrobial therapy. The isolate was sent to our local reference laboratory for antimicrobial susceptibility testing and to the National Microbiology Laboratory (NML; Winnipeg, Manitoba, Canada) for serogroup typing.

Peripheral blood cultures were reassuringly negative. Lumbar puncture was performed post-administration of empiric antibiotics. His cerebrospinal fluid (CSF) studies were reassuring with white blood cell count of 1×10^6^ l^−1^ and erythrocytes<500×10^6^ l^−1^ (normal range for both: 0–5×10^6^ l^−1^). Protein and glucose were normal at 0.34 gl^−1^ (0.15–0.45 gl^−1^) and 3.7 mmol l^−1^ (2.3–4.1 mmol l^−1^), respectively. The Gram stain was negative for polymorphs or organisms, with negative CSF culture. Trans-thoracic echocardiogram was performed to assess for potential septic emboli as the cause of his ocular manifestations, which was reassuringly negative for valvular lesions or vegetations suggestive of infective endocarditis. CT (computed tomography) of head (including orbit and paranasal sinuses) revealed moderate right maxillary sinus thickening without evidence of extraocular involvement or optic nerve involvement, suggesting his maxillary sinusitis being the introducing source of infection.

In terms of his blood-borne infection screening, he was human immunodeficiency virus and hepatitis C virus negative. His syphilis screening enzyme immunoassay was negative. He was hepatitis A virus immune and hepatitis B virus (HBV) non-immune with no evidence of acute HBV infection. Immunodeficiency and complement workup were also conducted, given the presence of invasive meningococcal infection (albeit in vitreous fluid only). However, there were no abnormal markers: his complement C3 was 1.17 (0.80–1.80 gl^−1^) and complement C4 was 0.16 (0.12–0.36 gl^−1^). Immunophenotyping analysis did not show any abnormal fractions for his CD3, CD4 and CD19 count, only a mildly elevated CD8 fraction of 42% (normal 10–40%); absolute counts of all those relative immune cells did not show any abnormalities and his CD4/CD8 ratio was within normal limits at 0.83. His antinuclear antibody ELISA was equivocal at 0.8 (negative<0.7), whereas his proteinase 3, anti-smooth muscle antibody, anti-mitochondrial antibody and myeloperoxidase antibodies were all negative. He did have a mildly elevated alpha-1-antitrypsin at 2.70 gl^−1^ (0.90–2.00 gl^−1^), although this is of unclear significance amidst his clinical presentation. Finally, protein electrophoresis noted some elevation in his gamma globulin levels (16.1 gl^−1^; normal 5.1–15.0 gl^−1^) but there was no monoclonal band on further analysis.

Antibiotic susceptibility testing confirmed MIC values of 0.002 μg/mL (ceftriaxone), 0.004 μg/mL (ciprofloxacin), 0.032 μg/mL (penicillin) and 0.008 μg/mL (rifampin), all of which were susceptible per Clinical and Laboratory Standards Institute M100 breakpoints [3]. After confirmed identification of *N. meningitidis* and no objective evidence of meningitis as detailed above (only maxillary sinusitis without intra-orbital or intra-cranial involvement), vancomycin was discontinued and the twice-daily ceftriaxone was adjusted to once-daily dosing to complete the 7-day treatment course. The isolate was subsequently confirmed by NML to be *N. meningitidis* serogroup Y.

The patient was closely monitored by the ophthalmology service. Anterior segment inflammation OD gradually improved, but a fundus view remained unobtainable. At 1 month from initial presentation, clinical examination revealed extensive posterior iris synechiae and a white cataract. B-scan ultrasound suggested a retinal detachment. The patient underwent cataract extraction and pars plana vitrectomy with silicone oil injection in March 2023. Intraoperatively, dense vitritis was noted, but the retina appeared flat in all quadrants. Due to excessive capsular adhesion to the iris, the lens capsule was removed and the patient was left aphakic. On post-op day 1, dense vitreous haemorrhage obstructed the view to the fundus. As this cleared, subretinal macular haemorrhage was identified. At 2 months post-op, the patient was unfortunately found to have proliferative vitreoretinopathy with an inferior tractional retinal detachment. He was taken back to the operating room for a second procedure, including pars plana vitrectomy, inferior retinectomy, endoscopic laser and silicone oil injection. The retina appeared flat with good oil fill in the immediate post-operative period, although visual prognosis remained guarded. Three weeks later, the patient developed a funnel retinal detachment, which was deemed inoperable. At his most recent follow-up visit, 1 year after initial presentation, best corrected visual acuity OD was hand motion and early phthisis was noted. Examination OS remained within normal limits.

## Discussion

**Fig. 1.**
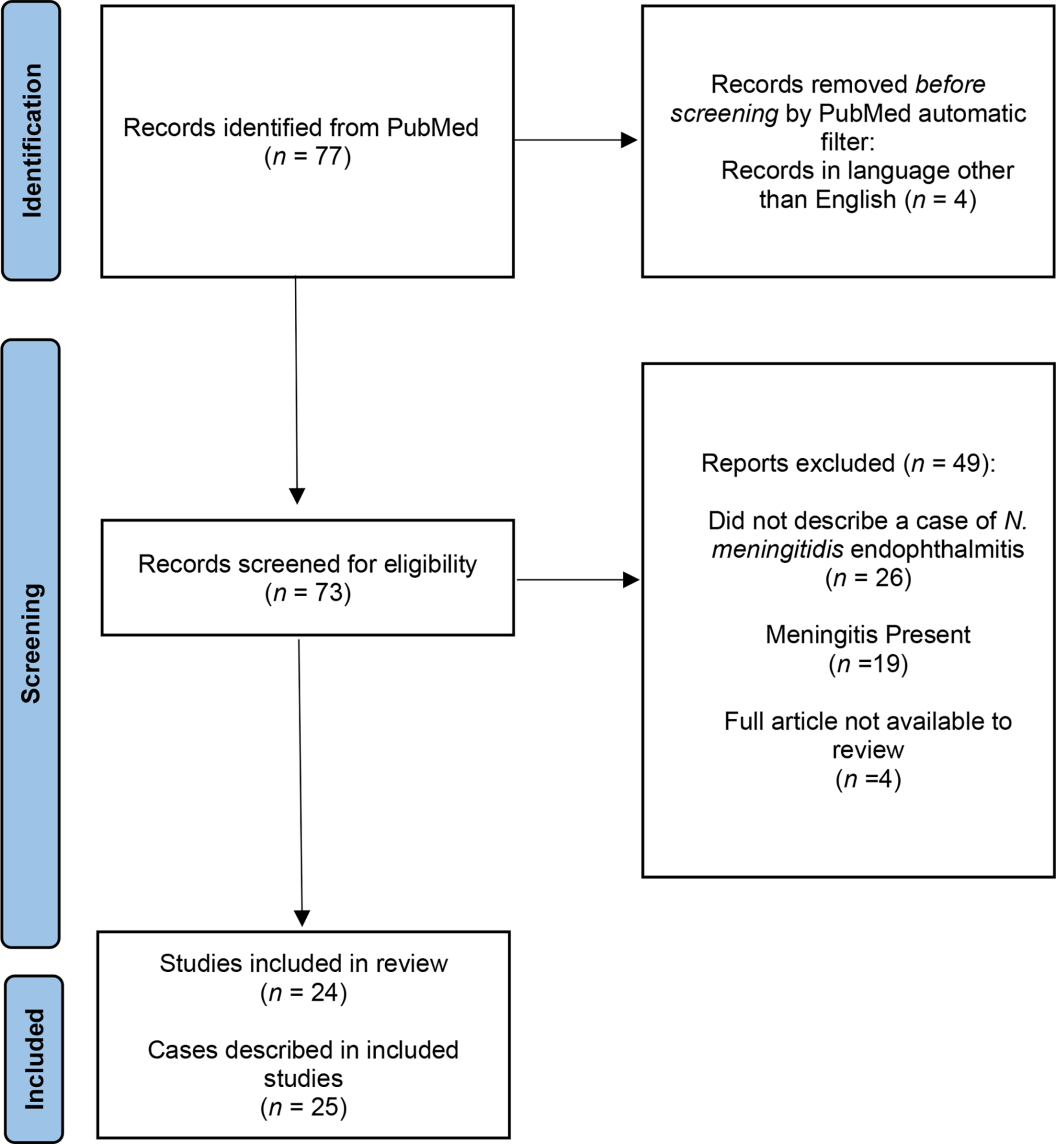
Flowchart of literature review. The search phrase used was ‘(Neisseria OR Meningococcal) AND endophthalmitis’.

We conducted a comprehensive literature review using the PubMed database to identify and characterize other cases of *N. meningitidis* endophthalmitis without evidence of meningitis (Fig. 1). Our inclusion criteria were articles describing cases of endophthalmitis caused by *N. meningitidis* written in English, available full articles with primary case data, and without documented meningitis. Using ‘(Neisseria OR meningococcal) AND endophthalmitis’ as our search phrase, we identified 77 articles from 1947 to 2024, of which 24 articles describing 25 cases met our criteria [4,27] (Tables S1 and S2, available in the online Supplementary Material). The identical search strategy was repeated on MEDLINE OVID but did not yield any additional cases.

The median age was 17, with patients ranging from within the first year of life to 82 years old (Table 1). In total, 13 cases (52%) occurred in paediatric patients. In total, 15 patients were male (60%) and most patients were healthy prior to infection (76%). We identified four cases of exogenously acquired infection, all of which involved eyes with pre-existing filtering blebs from previous surgeries acting as entry portals [11,19, 21, 26]. The rest of the cases were presumed endogenously acquired endophthalmitis, although there were only five with confirmed bacteraemia. While we excluded cases involving meningitis, there were other systemic manifestations of *N. meningitidis*, including fever, arthralgia and myalgia and rash, as well as more serious complications, including bacteraemia, pericarditis, septic arthritis and disseminated intravascular coagulation. All patients presented with a combination of red, painful eyes and decreased vision. Symptom onset was an average of 3 days prior to presentation. In total, 5 patients presented with bilateral infections, while 20 presented with a single affected eye. In total, 19 patients presented with a visual acuity of counting fingers or worse.

After presenting, 12 patients were initially diagnosed with something other than endophthalmitis (48%), such as uveitis, conjunctivitis or retinoblastoma. Patients who were diagnosed with something other than endophthalmitis received antibiotics an average of 4.0±4.9 days after presenting to hospital or to an ophthalmologist, while those diagnosed on initial presentation received urgent systemic or intravitreal antibiotic treatment. On average, patients received systemic or intravitreal antibiotics 4.6±4.8 days after ocular symptoms began. All patients eventually received systemic antibiotics, most commonly third-generation cephalosporins (16, 64%). Other antibiotics used included penicillin, ampicillin, fluoroquinolones and gentamicin. The total duration of systemic antibiotics was either not stated (12, 48%), ≤2 weeks (9, 36%) or>2 weeks (4, 16%). In total, 18 patients received intravitreal antibiotics as well (72%), most commonly ceftazidime and vancomycin (9 cases, 36%). Other intravitreal antibiotics included amikacin, chloramphenicol and penicillin. In total, 18 patients also received topical antibiotics (72%), while 5 patients received subconjunctival antibiotics. Subconjunctival antibiotics have questionable efficacy in the treatment of endophthalmitis [28,29], and it is notable that the five articles detailing subconjunctival antibiotic use were published in 2003 or earlier [4,10, 15, 23, 26]. Steroids of various routes were used in 17 patients (68%), with 6 patients receiving topical steroid alone, 1 patient receiving intravitreal steroid alone and 10 patients receiving combinations of topical, systemic, intravitreal or subconjunctival steroids. Mydriatics were used in 16 patients (64%). Vitrectomy or other surgical procedures, including lensectomy, were pursued in 16 patients (64%).

In terms of outcomes, only 10 of the 25 patients had visual acuity of >20/200 in at least one of their affected eyes at last noted follow-up, including 3 patients with full recoveries. One patient had a visual acuity of less than counting fingers at baseline, while the other 14 were left with visual acuity<20/200. This includes two patients who underwent enucleation and eight who were left with light perception or worse. Breakdowns between these relatively ‘functional’ and ‘non-functional’ outcomes are detailed in Table 2. Briefly, the median age of patients with functional outcomes was younger (13 versus 19.5), but there was no clear difference in comorbidities, presenting visual acuity, presence of steroid use or surgical procedures. In total, 6 of the 10 (60%) patients with functional outcomes were correctly diagnosed with endophthalmitis at initial presentation compared to 6 of the 14 (42.9%) patients with non-functional outcomes. There was no clear difference between the time from ocular symptom onset to antibiotic use between the two groups; however, it is possible that delayed treatment was a factor in several cases. On average, the functional outcome group received antibiotics 1.1±1.6 days after presentation, compared to the non-functional outcome group with 2.5±4.9 days after presentation. However, this was not uniform across all cases, and even a case in which antibiotics were delayed for 5 days had a functional outcome [16], while several cases in which antibiotics were administered immediately had non-functional outcomes (6/13, 46.2%). Additionally, longer durations of systemic antibiotic therapy did not seem to have any impact on outcomes. Of the five functional cases that reported on antibiotic duration, all five were treated for up to 2 weeks. Meanwhile, of the eight non-functional cases with antibiotic duration reported, four were treated for longer than 2 weeks, while four were treated for 2 weeks or less.

*N. meningitidis* was ultimately detected in all cases, including from vitreous samples (16, 64%), anterior chamber samples (3, 12%), blood (5, 20%) and skin scrapings (1, 4%). The organism was recovered by culture in 18 cases (72%), a combination of culture and PCR in two cases (8%), PCR alone in four cases (16%) and in one case was ultimately diagnosed via 16S rRNA (4%) [14]. In another case, vitreous culture was negative despite Gram-negative diplococci on Gram stain, and *N. meningitidis* was ultimately detected via PCR from skin scrapings [12].

Serogroup was reported in 12 cases, with 10 cases of serogroup C and 1 case each of serogroups Y and W135. This serogroup C predominance differs compared to another eye disease caused by *N. meningitidis*, primary meningococcal conjunctivitis, which is more commonly associated with serogroups B or A [30,31]. Of note, the isolate in our case was determined to be serogroup Y. There have been increased cases of *N. meningitidis* serogroup Y causing invasive meningococcal disease in North America. As per the Centers for Disease Control and Prevention epidemiological data, there were 422 reported serogroup Y cases in 2023, the highest annual count within the past decade [32]. Of cases with sequencing data available, 68% (101 of 148) were due to a specific meningococcal strain [sequence type (ST)-1466]. Interestingly, most cases presented atypically without meningitis (64% presented with bacteraemia and at least 4% presented with septic arthritis). Thus far, serogroup Y ST-1466 isolates are fully susceptible to first-line antibiotics used for treatment and prophylaxis of meningococcal disease, although ciprofloxacin resistance has been reported in some serogroup Y strains that have a disproportionate impact on Hispanic populations in the USA [33]. Given this shift in atypical clinical presentations for meningococcal disease, there should be a lower threshold of suspicion, especially for vulnerable populations or those with unclear vaccination status.

Overall, endophthalmitis is a rare complication of *N. meningitidis* infection. A review by Paez Guillan *et al.* identified 37 cases in the past 20 years and found that it is generally accompanied by meningitis in three-quarters of cases [34]. It is unclear currently how our patient acquired his infection. In our literature review, we identified four cases of exogenously acquired *N. meningitidis* endophthalmitis; however, all four featured eyes had pre-existing filtering blebs acting as portals for ocular infection. Alternatively, the other 20 cases of presumably endogenously acquired endophthalmitis featured infectious prodromes and systemic symptoms. Our patient had no history of ocular procedures but did have a short course of diarrhoea prior to ocular symptom onset. Unfortunately, endophthalmitis can have a poor prognosis regardless of prompt recognition and intervention, as in our patient.

## supplementary material

10.1099/acmi.0.000901.v3 Uncited Supplementary Material 1.

10.1099/acmi.0.000901.v3 Uncited Supplementary Material 2.

## Abbreviations

ALP: alkaline phosphatase
ALT: alanine transferase
CSF: cerebrospinal fluid
CT: computed tomography
eGFR: estimated glomerular filtration rate
GGT: gamma-glutamyl transferase
HBV: hepatitis B virus
MCV: mean corpuscular volume
MIC: minimum inhibitory concentration
NML: National Microbiology Laboratory
PO: per os
ST: sequence type
