# Supplementary material for: Isolated Neisseria meningitidis-associated endophthalmitis in an immunocompetent host: case report and literature review
Source: Access Microbiol. 2025 Mar 7;7(3):000901.v3. doi: 10.1099/acmi.0.000901.v3 (PMC11936350; doi:10.1099/acmi.0.000901.v3)
Supplement: Uncited Supplementary Material 1. [file acmi-7-00901-s001.pdf]

Supplementary Table 1: Summary of *Neisseria meningitidis* endophthalmitis cases, clinical presentation and diagnosis, management.

Ref #	Sex	Age (Years)	Comorbidities	Systemic symptoms	Presenting visual acuity in affected eye	Time from presentation to empiric antibiotics	Antibiotic route	Detection Source, Method	Serogroup	Surgical Procedure (if required)	Final Visual Acuity
(13)	F	7	None	Fever, rash, V/D	LP RE, CF LE	Same day	IV	Blood, culture	NR	None	20/20 OU
(18)	M	2	None	Fever, Rash, cough, arthralgia	Not stated	Same day	IV, T	Blood, culture	C	None	Full recovery
(26)	M	53	AUD, liver disease, COPD, filtering bleb after cataract extraction	None	HM	Same day	IV, IVT, SCJ, T	Vitreous, culture	NR	None	20/40
(6)	M	1	None	Fever, rash	Not stated	4 days	IV, IVT, T	Vitreous, culture	C	Vitrectomy	Poor
(7)	F	58	None	Fever, Pericarditis	CF LE, 20/20 RE	Same day	IV	Blood, culture	C	Vitreolensectomy	LP OS, 20/200 OD
(15)	M	23	None	Fever, arthralgia, sore throat	LP	3 days	IV, SCJ, T	Aqueous, culture	C	Vitrectomy	LP
(4)	M	19	None	V/D, myalgia	HM	3 days	IV, SCJ, T	Blood, culture	C	None	20/40
(19)	F		Filtering Bleb	None	LP	Same day	IV, IVT, T	Vitreous, culture	NR	Vitrectomy	20/100
(24)	M	0	None	Fever, Rash	No LP	Same day	IV	Vitreous, culture	NR	Enucleation	
(25)	M	17	None	Rash, myalgia, arthralgia	HM	2 days	IV, IVT, T	Vitreous, culture	NR	Vitrectomy	LP

(21)	M	81	Filtering bleb post-cataract extraction	None	LP (Baseline CF)	Same day	IV, IVT, T	Vitreous, culture	Y	Vitrectomy	HM
(17)	M	16	None	Rhinitis, headache	20/150	2 days	IV, IVT, T	Vitreous + Aqueous, culture	NR	Vitrectomy	Minimal
(23)	F	2	None	Rash, lethargy -> multiorgan failure, DIC	Not stated	Same day	IV, IVT, SCJ	Blood, culture	NR	Vitrectomy	20/40 OD, 20/70 OS
(9)	F	54	HTN	V/D, arthralgia	20/80	1 day	IV, IVT, T	Vitreous, culture	NR	Vitrectomy	LP
(10)	F	13	None	Fever, rash, arthralgia	LP BE	1 day	IV, IVT, SCJ, T	Vitreous, PCR	C	Vitrectomy	LP OS, 20/100 OD
(16)	M	17	None	Sore throat, arthralgia	CF	5 days	IV	Aqueous, culture + PCR	C	Vitrectomy	20/20
(12)	F	7	None	Fever, rash, headache, neck stiffness (transient)	Not stated	Not stated	IV, IVT	Vitreous, gram stain positive. PCR, skin scrapping	C	None	LP
(27)	M	16	None	Fever, arthralgia, rash, testicle swelling	HM	Same day	IV, IVT	Vitreous, culture	NR	Multiple (not stated)	Poor
(8)	M	27	None	Malaise, rash, arthralgia	CF RE, 20/40 LE	Same day	IV, IVT, T	Vitreous + Blood, culture	C	None	20/60 OD, 20/20 OS

(5)	F	29	None	Fever, V/D, myalgia, rash	LP	Same day	IV, IVT	Vitreous, culture	NR	Vitrectomy	CF
(14)	F	4	None	Croup -> fever, rash	HM	19 days	IV, IVT, T	Aqueous, 16s rRNA	C	Enucleation	
(22)	M	24	None	Malaise, Septic Arthritis	LP	Same day	IV, IVT, T	Vitreous, culture + PCR	NR	Lensectomy	LP
	F	22	Diabetes	Malaise, Septic arthritis	LP	Same day	IV, IVT, T	Vitreous, PCR	W135	None	No LP
(11)	M	81	AFib, HTN, dyslipidemia, multiple previous eye surgeries with filtering bleb	None	No LP	Same day	IV, IVT, T	Vitreous + conjunctiva, culture	NR	Vitrectomy	20/400
(20)	M	9	None	Malaise, V/D	LP	2 days	IV, IVT, T	Vitreous, PCR	NR	Vitrectomy	20/25

16s rRNA 16s Ribosomal ribonucleic acid sequencing, AFib Atrial Fibrillation, AUD Alcohol Use Disorder, CF Counting Fingers, COPD Chronic Obstructive Pulmonary Disease, DIC Disseminated Intravascular Coagulation, F female, HM Hand motions, HTN Hypertension, IV intravenous, IVT Intravitreal, LP Light perception, M male, NR Not Reported, OD Oculus Dexter, OS Ocular Sinister, OU Ocular Uterque, PCR Polymerase Chain Reaction, SCJ Subconjunctival, T topical, V/D vomiting/diarrhea
